# Empowering mothers as household health workers

**DOI:** 10.1186/1753-6561-6-S5-O28

**Published:** 2012-09-28

**Authors:** Tage Kanno, T Taloh, Dimong Padung

**Affiliations:** 1 Future Generations Arunachal, Itanagar, India; 2 Joint Director (Family Welfare), Government of Arunachal Pradesh, Itanagar, India; 3 Nodal Officer (National Rural Health Mission), Government of Arunachal Pradesh, Itanagar, India

## Introduction

Physical inaccessibility and the lack of awareness have remained major constraints to health service delivery in Arunachal Pradesh. Inadequate trained human resource for health aggravates the situation. Road density in Arunachal Pradesh in 2001 was only 17.3 kilometer per 100 square kilometer of area as against the national average of 82 kilometer. This results in nearly 26.5% of the population living with no road connectivity at all [1]. The health infrastructure development has been slow and service delivery remains highly constrained. This has been aggravated by presence of total of only 700 doctors and 900 nurses, who too are unwilling to serve in remote areas.

The Government of Arunachal Pradesh started the Public Private Partnership (PPP) project as a part of the National Rural Health Mission in 2006 that outsourced the management of one Primary Health Center (PHC) in each of the 16 districts to non-governmental organizations. The one such PHC situated at the Sille in the East Siang district is being managed by the Future Generations Arunachal. A pilot project entitled Mothers’ Training was carried out from May 2009 to April, 2011 with the premise that empowering the mothers to take care of their own health has the potential to dramatically improve the health status of the communities in inaccessible parts of Arunachal Pradesh. The idea was to train one woman from each household in the catchment areas of the PHC. Each month, one woman from each of the six main villages in the PHC catchment area was selected and was trained on basic home-based health care for a month. The training method consisted of one-hour talk on health issue everyday and involving the trainees in the works in the PHC the rest of the day.

## Methods

The outcome of the above-described project was measured with various health indicators captured at the PHC.

## Results

Dramatic improvements in health care indicators, especially those related to health behavior, were noted. Outpatient attendance dropped from 10,543 in 2008 to 6,838 in 2011. Similarly, total antenatal registration dropped from 269 in 2008 to 149 in 2011. However, percentage of pregnant women registering themselves in the antenatal clinics in the first trimester increased from 39.4% in 2008 to 69.1% in 2011. There was also an increase in institutional deliveries from 33.8% in 2008 to 44.9% in 2011. In 2008, 5,742 slides were collected for tests for malarial parasite whereas it was only 2,122 in 2011. No death due to malaria has been reported whereas the disease was a major cause of mortality in the area earlier.

Since posting of qualified personnel in the remote areas has been a major hurdle to health service delivery in Arunachal Pradesh, it is recommended that the existing health facilities like PHCs, the community health centers, and the district hospitals be used as community training centers on health so that they can participate in the health care delivery of the government. This will not only improve the health status of the communities but also imbibe a sense of ownership in the health care process. Since the existing infrastructure and manpower will be utilized, this can be accomplished with minimal additional resource inputs.

## Competing interests

Authors declare that they have no conflict of interest.

## Funding statement

This abstract draws on a Public Private Partnership project funded by the National Rural Health Mission through the Government of India and the Government of Arunachal Pradesh, with additional funding by the Future Generations, USA for Mothers’ Training program.
